# Weighted Gene Co-expression Network Analysis of Endometriosis and Identification of Functional Modules Associated With Its Main Hallmarks

**DOI:** 10.3389/fgene.2018.00453

**Published:** 2018-10-12

**Authors:** Mohammad Reza Bakhtiarizadeh, Batool Hosseinpour, Maryam Shahhoseini, Arthur Korte, Peyman Gifani

**Affiliations:** ^1^Department of Animal and Poultry Science, College of Aburaihan, University of Tehran, Tehran, Iran; ^2^Department of Agriculture, Iranian Research Organization for Science and Technology, Tehran, Iran; ^3^Department of Genetics, Reproductive Biomedicine Research Center, Royan Institute for Reproductive Biomedicine, ACECR, Tehran, Iran; ^4^Center for Computational and Theoretical Biology, University of Würzburg, Würzburg, Germany; ^5^Cambridge Systems Biology Centre, Department of Genetics, University of Cambridge, Cambridge, United Kingdom; ^6^AI VIVO Ltd., St. John’s Innovation Centre, Cambridge, United Kingdom

**Keywords:** endometriosis, module, weighted gene co-expression network, hub genes, expression

## Abstract

Although many genes have been identified using high throughput technologies in endometriosis (ES), only a small number of individual genes have been analyzed functionally. This is due to the complexity of the disease that has different stages and is affected by various genetic and environmental factors. Many genes are upregulated or downregulated at each stage of the disease, thus making it difficult to identify key genes. In addition, little is known about the differences between the different stages of the disease. We assumed that the study of the identified genes in ES at a system-level can help to better understand the molecular mechanism of the disease at different stages of the development. We used publicly available microarray data containing archived endometrial samples from women with minimal/mild endometriosis (MMES), mild/severe endometriosis (MSES) and without endometriosis. Using weighted gene co-expression analysis (WGCNA), functional modules were derived from normal endometrium (NEM) as the reference sample. Subsequently, we tested whether the topology or connectivity pattern of the modules was preserved in MMES and/or MSES. Common and specific hub genes were identified in non-preserved modules. Accordingly, hub genes were detected in the non-preserved modules at each stage. We identified sixteen co-expression modules. Of the 16 modules, nine were non-preserved in both MMES and MSES whereas five were preserved in NEM, MMES, and MSES. Importantly, two non-preserved modules were found in either MMES or MSES, highlighting differences between the two stages of the disease. Analyzing the hub genes in the non-preserved modules showed that they mostly lost or gained their centrality in NEM after developing the disease into MMES and MSES. The same scenario was observed, when the severeness of the disease switched from MMES to MSES. Interestingly, the expression analysis of the new selected gene candidates including CC2D2A, AEBP1, HOXB6, IER3, and STX18 as well as IGF-1, CYP11A1 and MMP-2 could validate such shifts between different stages. The overrepresented gene ontology (GO) terms were enriched in specific modules, such as genetic disposition, estrogen dependence, progesterone resistance and inflammation, which are known as endometriosis hallmarks. Some modules uncovered novel co-expressed gene clusters that were not previously discovered.

## Introduction

During the menstrual cycle, a cycle of changes occurs in both the uterus and ovary. During this cycle, the cyclical changes in normal endometrium (NEM) are divided into three phases: (i) menstrual phase, (ii) proliferative phase, and (iii) secretory phase. Normally, endometrial glandular and stromal tissues grow inside the uterus but in some condition, they grow outside the uterus, which is known as the endometriosis (ES) disease. This extrauterine growth (ectopic endometrium) induces a chronic, inflammatory reaction. While some women with ES show painful symptoms and/or infertility, others experience no pain at all. It is a chronic estrogen-dependent gynecological disease and affects 6–10% of reproductive-age women but its frequency in women with pain and/or infertility is as high as 35–50% (Meuleman et al., 2009). Using laparoscopy, ES is diagnosed and staged into four classes based on the level of severity and progression of the disease: stage I (minimal), stage II (mild), stage III (moderate), and stage IV (severe). On average, it takes about 11 years from symptom onset to diagnosis. Thus, there is still an urgent need for minimally invasive diagnostic tests (Fassbender et al., 2015), which requires deeper views on molecular mechanism of the disease development. Although many researchers attempt to find key regulators of the disease, a few numbers of genes have been functionally analyzed. This is mainly due to the complexity of the disease, which makes it difficult for researchers to study its underlying molecular mechanism.

Endometriosis is characterized by a series of molecular hallmarks that include genetic predisposition, estrogen dependence, progesterone resistance and inflammation. Many previous studies provided molecular evidence for these hallmarks (Burney and Giudice, 2012) and focused on identifying key genes controlling these characteristics. Several large-scale gene expression studies investigated differentially expressed genes between NEM and ES across the menstrual cycle of women with mild/moderate severe ES (Kao et al., 2003; Matsuzaki et al., 2005; Sherwin et al., 2008; Tamaresis et al., 2014). Kao et al. (2003) and Matsuzaki et al. (2005) identified a large number of genes in the early and mid-secretory phases of the menstrual cycle, whose endometrial expression differed between controls and patients with ES. Sherwin et al. (2008) identified a few differentially expressed transcripts, in the late secretory eutopic endometrium, between women with and without ES. Moreover, they found that no transcripts were differentially expressed between minimal/mild and moderate/severe ES. A number of genes implicated in ES were identified including BCL-2 (Jones et al., 1998), NF- κB (González-Ramos et al., 2010), TGF-β (Oosterlynck et al., 1994), TNF-α (Eisermann et al., 1988), CYP19 (Noble et al., 1996), IL-6 (Harada et al., 1997), 17βHSD-2 (Zeitoun et al., 1998), MMP3,7 (Bruner-Tran et al., 2002), KRAS (Dinulescu et al., 2005), PTEN (Dinulescu et al., 2005), and PGE2 (Badawy et al., 1984).

Since ES is affected by a series of molecular hallmarks, classification and attribution of the identified genes only based on gene expression analyses are impossible. In addition, different stages of the disease were classified using the visible symptoms; however, little is known about the molecular mechanisms underlying each stage of the disease. Thus, we hypothesized that the study of the differentially expressed genes in ES at a system level may give novel information about the contribution of the genes to the ES development.

Gene co-expression network analysis has been used to extract new information using differentially expressed genes. The aim of this analysis is to analyze gene co-expression network to classify sets of coordinately expressed genes into a number of modules. These modules rely on the assumption that strongly correlated expression levels of a group of genes are likely to be functionally associated. Weighted gene co-expression network analysis (WGCNA) is one of the most widely used methods (Horvath, 2011), which is particularly well suited for constructing gene co-expression networks in this study.

In WGCNA, the direct and indirect relationships between the genes are measured and interpreted as connectivity. Highly correlated genes are grouped into large modules (or co-expression modules) based on similarities in their expression profiles. Modules are often enriched for genes that share similar biological functions. Therefore, different modules are often involved in specific biological functions. This method not only considers the co-expression patterns between genes, but also allows the identification of highly connected genes (Zhang and Horvath, 2005; Langfelder and Horvath, 2008). WGCNA measures intramodular gene connectivity and, highly connected genes are defined as hub genes. These hub genes are centrally located in their respective modules and may thus be playing critical roles in the clinic trait.

We can also determine whether the modular structure is reproducible and preserved in another dataset. For instance, by this approach, it is possible to determine whether a module found in one dataset (normal samples) can also be found in another dataset (disease samples) (Horvath, 2011). Thus, some modules and their hub genes that are not preserved between samples (normal vs. disease) may be involved in pathological processes. This hypothesis was successfully applied in previous studies (Mukund and Subramaniam, 2015; Medina and Lubovac-Pilav, 2016).

Weighted gene co-expression analysis has been successfully used to identify highly connected hub genes and significant modules in various cancer types (Udyavar et al., 2013; Yang et al., 2014), murine embryonic stem cells (Mason et al., 2009), coronary artery disease (Liu et al., 2016) and major depressive disorder (Malki et al., 2013). Using WGCNA, Yang et al. constructed gene co-expression networks for multiple cancer types and found that some prognostic modules were conserved across different cancer types (Yang et al., 2014). They also predicted that prognostic genes significantly tended not to be hub genes. Instead, predicted hub genes were highly specific for each cancer and showed a little overlap. Xu et al. (2016) performed WGCNA to predict significant modules and hub genes in hepatocellular carcinoma and identified eight distinct modules and several potential biomarkers.

Our main assumption in this study was to discover and analyze non-preserved modules between NEM, MMES, and MSES to improve our understanding of the ES regulatory mechanism. This can potentially lead to novel insight into drug target discovery with future clinical implications in addition to exploring new potential diagnostic and prognostic biomarkers. Based on this assumption, we aimed to answer a number of questions: (1) what are significant modules at different stages of ES? (2) which genes tend to be hubs in significant modules? (3) what are common hub genes in NEM and ES? (4) what are specific hub genes in NEM and ES? (5) which significant modules are topologically preserved at each stage of the disease? (6) which GO terms are enriched in each module? and (7) which of the previously identified genes are rediscovered as hubs in the modules, and where are they in the network?

## Materials and Methods

### Datasets

Raw CEL files of GSE51981 (Tamaresis et al., 2014) were obtained from the Gene Expression Omnibus (GEO) database at the National Center for Biotechnology Information (NCBI), which were based on the platform of GPL570 Affymetrix Human Genome U133 Plus 2.0 Array. The data contained archived endometrial samples from women with minimal/mild ES (*n* = 27), mild/severe ES (*n* = 48) and without ES (*n* = 34). Women were 20–50 years old without hormonal treatment within previous 3 months and presence of malignancy or major systemic diseases.

### Microarray Data Analysis and Preprocessing

A total of 109 raw samples were simultaneously normalized using R package for GCRMA. Expression values were log_2_-transformed before being further processed. Empirical Bayes moderated *t*-statistics from the Bioconductor package limma (linear model for microarray data) was used to identify DEGs between NEM vs. MMES and NEM vs. MSES. Using limma, a linear model was fitted to expression data for each gene (Smyth, 2005). Probe sets with fold change (FC) ≥ 1.5 and false discovery rate < 0.05 were determined as DEGs and selected for further analysis. Then, only common DEGs between the two comparisons (NEM vs. MMES and NEM vs. MSES) were used for gene co-expression network analysis.

In the next step, the probe sets were mapped to respective gene symbols using the array annotation data hgu133plus2.db. The expression of a given gene is usually measured by multiple probes; therefore, the probe sets mapped to multiple genes were discarded. The collapseRows function of the WGCNA package was applied to collapse the multiple probes corresponding to the same gene. The effectiveness of this method was reported in a previous study (Miller et al., 2011).

Gene co-expression analyses are very sensitive to the presence of outliers It was demonstrated that distance-based networks (or sample networks) were useful for detecting outlying samples or observations (Horvath, 2011). Thus, the adjacency function in the WGCNA package was applied to calculate distance-based adjacency matrices for identifying outlying samples. Samples with a standardized connectivity score of less than -2.5 were removed (Horvath, 2011). Then, we used the *goodSamplesGenes* function of the WGCNA package to iteratively remove samples and genes with too many missing entries (default = more than 50% missing entries) and genes with zero variance.

### Construction of a Signed Weighted Gene Co-expression Network

Based on the assumption that non-preserved modules between NEM and ES are important, normal samples were considered as the reference set for module derivation and the other two datasets (MMES and MSES) were used for module reproducibility. Using WGCNA, we created a signed weighted gene co-expression network based on normal gene expression data. In unsigned correlations, positively and negatively correlated genes (activation or repression effects of genes) are grouped into the same cluster and cannot be discriminated (Mason et al., 2009). In contrast, the advantage of applying a signed network is that it considers the sign of the underlying correlation coefficient. Moreover, it has been shown that signed networks can identify modules with more significant enrichment of functional groups (Horvath, 2011). Briefly, an adjacency matrix was created from the pairwise biweight midcorrelation coefficients between all genes, as this correlation method is often more powerful than Spearman correlation and more robust than Pearson correlation (Song et al., 2012).

Approximate scale free topology is a fundamental property of biological gene networks, in which some genes are more connected than others (hub genes). Therefore, the adjacency matrix was replaced with the weighted adjacency matrix by raising the correlations to the power of 13, which was chosen using the scale-free topology criterion (Horvath, 2011). We selected the power for which scale-free topology fitting index (*R*^2^) was ≥0.8 by plotting the *R*^2^ against soft thresholds (power β). **Supplementary Figure S1** presents the relationship between the β and *R*^2^. Subsequently, the weighted adjacency matrix was transformed into a topological overlap matrix (TOM) and the corresponding dissimilarity was calculated to minimize effects of noise and spurious associations. The TOM measures connectivity of a pair of genes in relation to all other genes in the network (network interconnectedness). Higher TOM values show that a pair of genes is more likely to be connected to each other and to a shared set of genes. The TOM measure, therefore, allows to identify gene modules whose members share strong interconnectivity patterns as we are able to create more robust co-expression relationships (Horvath, 2011). Each TOM was then used as input for average linkage hierarchical clustering (by defining a dissimilarity matrix, 1-TOM) and the modules were identified in the resulting dendrogram through a dynamic hybrid tree cutting algorithm. Finally, the modules with highly correlated eigengenes were merged, as the minimum height for merging modules was set to 0.25 (Horvath, 2011).

### Preservation Analysis

To assess the preservation levels of normal network modules in the ES datasets, the *modulePreservation* function in the WGCNA package was applied using two network based composite preservation statistics (*Zsummary* and *medianRank*). We applied Zsummary to investigate significance of module preservation and medianRank to detect module preservation using permutation testing (200 permutations). Zsummary combines different preservation statistics into a single overall measure of preservation, which are equally important for judging the preservation of a network module. Zsummary also investigates whether modules identified in the normal dataset remain highly connected in the ES datasets (density) and whether node connections between the genes are similar between the normal and ES datasets (connectivity). The higher the value of a Zsummary, the stronger the evidence that the module is preserved in a certain condition/treatment. However, Zsummary shows a strong dependence on module size (as tends to increase with increase in module size). Therefore, it is more significant to observe that the connectivity patterns among hundreds of nodes are preserved than to observe the same among say only six nodes. However, sometimes, we need to compare preservation statistics of modules of different sizes. In this case, medianRank can be used. The medianRank is based on the observed preservation statistics and shows no dependence on module size. In contrast with Zsummary, a module with a lower medianRank is more preserved than a module with a high medianRank (Horvath, 2011). In this study, by combining Zsummary and medianRank and based on the empirical thresholds proposed in previous studies (Langfelder et al., 2011), a module was considered as non-preserved if it had Zsummary < 5 or medianRank ≥ 8 (Horvath, 2011).

### Detection of Hub Genes

To identify highly connected genes or hub genes, the WGCNA package was used for calculating Eigengene-based module connectivity or module membership (*k*_ME_) measures for a particular gene within a given non-preserved module. The module membership can be determined by the correlation between the expression profile of a gene and the module eigengene (or first principal component) of a module. This measure quantifies how close a gene is to a given module. Therefore, it can be applied to detect module hub genes, as genes with high module membership are labeled as intramodular hub genes. Hub genes are representative of the module’s overall function and have a high likelihood to be critical components within the module. This measure was used to identify hub genes of non-preserved modules associated with different states (NEM, MMES, and MSES), as genes whose |*k*_ME_| was ≥0.7 were considered as hub genes to the respective module (Horvath, 2011).

### Functional Enrichment Analysis

Using GO analysis, the biological process ontology of the modules as well as their hub genes were investigated using the Enrichr tool (Chen et al., 2013). Co-expressed genes in some modules may be co-regulated by common TF(s) (Mason et al., 2009; Bakhtiarizadeh et al., 2014). Hereby, to identify potential common transcription factors that may control transcription of module genes, the “TRANSFAC_and_JASPAR_PWMs” section of the Enrichr tool (Chen et al., 2013) was applied. The corrected *p*-value (false discovery rate, FDR) < 0.05 was chosen to identify significant outcome.

Using GO analysis, the biological process ontology of the modules as well as their hub genes were investigated using the Enrichr tool.

The *R* script used in this study is available as **Supplementary File S1**.

#### Patients and Tissue Specimens

This study was approved by the Institutional Ethics Committee of the Royan Institute, and written informed consents were obtained before the collection of tissue samples. Endometrial biopsy specimens were collected from 16 ES patients. All the patients were 20–45 years old, consulting for infertility and/or pelvic pain, and found to have no endometrial hyperplasia or neoplasia. Eutopic biopsies were obtained with the use of Pipelle. Moderate to severe ES (the stages III–IV of the disease) was determined according to the revised classification of the American Fertility Society (Andrews et al., 1985).

Of the 16 participants, 8 had mild ES and 8 had severe ES. Eight normal endometrium samples during the menstrual cycle were tested as control group in this study. Participants in the control group taking part in this investigation were 20–40 years old with regular cycles, showed no evidence of any pathologic uterine disorder, and had not used oral contraception or an intrauterine device in the previous 3 months. Moreover, none of the participants in the control group had visible endometrial hyperplasia or neoplasia, inflammatory disease, or ES at the time of clinical examination or laparoscopy. The cycle day was determined according to the cycle history and histologic criteria. Each of the control women had at least one child by natural conception.

#### Real-Time Reverse-Transcription Polymerase Chain Reaction (Real-Time RT-PCR)

Total RNA was extracted separately from each group with the use of TRI reagent (Sigma) and treated with DNase I (Fermentas). First-strand cDNA synthesis was performed with the use of random hexamer primers and the superscript II reverse transcriptase system (Fermentas). For ensuring cDNA synthesis, the products were checked with the use of human β-actin as a housekeeping gene (Metabion) and platinum Blue PCR Super Mix (Invitrogen). Primer sequences used in this study are presented in **Supplementary File S2**.

Quantitative polymerase chain reaction (PCR) was performed on the prepared cDNA samples with the use of primers designed for matrix metallopeptidase 2 (MMP2), Homeobox B6 (HOXB6), cytochrome P450 family 11 subfamily A member 1 (CYP11A1), coiled-coil and C2 domain containing 2A (CC2D2A), immediate early response 3 (IER3), Syntaxin 18 (STX18), AE binding protein 1 (AEBP1), and insulin like growth factor 1 (IGF-1). Real-time PCR was performed under standard conditions, and all experiments were run in triplicate.

## Results

### Data Processing and DEGs Screening

In total, 109 arrays of GSE51981 (containing 34 NEM, 27 MMES, and 48 MSES) were used for identifying DGEs analysis. A total of 25,198 and 20,409 probe sets was found to be differentially expressed between NEM vs. MMES and NEM vs. MSES, respectively. Overall, 18,293 common probe sets were identified and mapped to 10,139 genes. After screening the outlier samples, four samples were discarded including two samples from MMES (GSM1256685 and GSM1256686) and two samples from MSES (GSM1256678 and GSM1256679). Finally, after other processing steps (removing the genes with missing values), 10,128 DEGs and 105 samples remained and served for network construction (**Supplementary File S3**).

### Identification of Modules Related to ES Development Using Signed WGCNA

**FIGURE 1:**
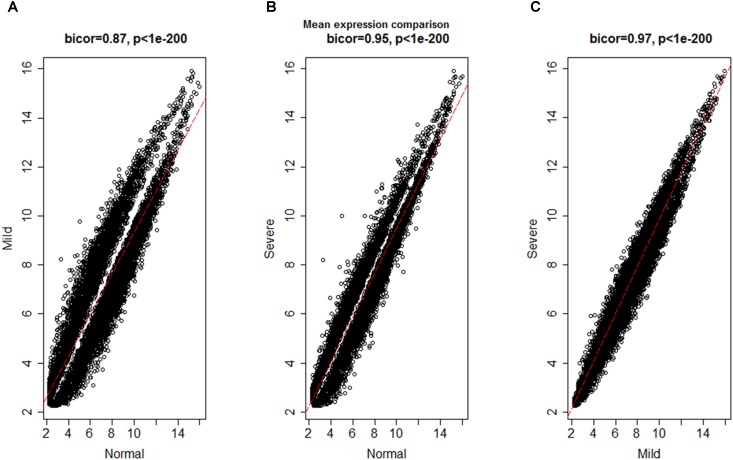
Biweight midcorrelation (bicor) between gene expression levels in normal endometrium and ES disease stages. Each dot corresponds to a gene and the red line represents to the best-fit regression line. Correlation coefficients are reported in respective graph. Gene expression patterns were strongly correlated between normal endometrium and ES stages **(A,B)** as well as between ES stages **(C)**.

**FIGURE 2:**
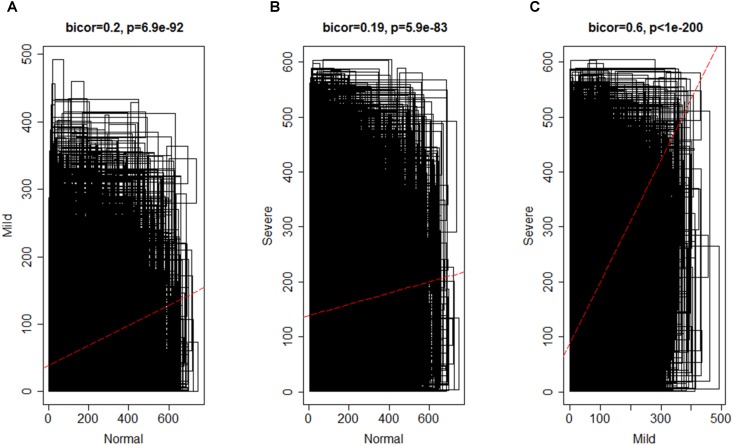
Biweight midcorrelation (bicor) between connectivity patterns in normal endometrium and ES disease stages; *x-* and *y-* axes denote the average number of connectivity of a gene and the red line represents to the best-fit regression line. Connectivity pattern was less preserved between NEM and ES stages **(A,B)** compared to MMES vs. MSES **(C)**.

To investigate the global differences in gene expression architecture, we compared mean expression and connectivity patterns between the NEM and ES samples using biweight midcorrelation. Gene expression patterns were strongly correlated between NEM and ES stages (MMES and MSES) as well as between MMES and MSES stages (**Figure 1**). However, connectivity pattern between the NEM and ES stages had lower density compared to the connectivity pattern between MMES and MSES stages (**Figure 2**).

The discrepancy between gene expression profile and weaker connectivity indicated that gene expression profile was similar in both conditions (NEM vs. ES), but the mode by which genes were interconnected was not so preserved. It is worth noting that connectivity pattern was similar in MMES vs. MSES. It suggests that ES development can be due to altered connectivity among genes instead of gene expression profiles. Therefore, it is valuable to use connectivity network as a complementary method to differential expression gene analysis for discriminating NEM from ES samples.

**FIGURE 3:**
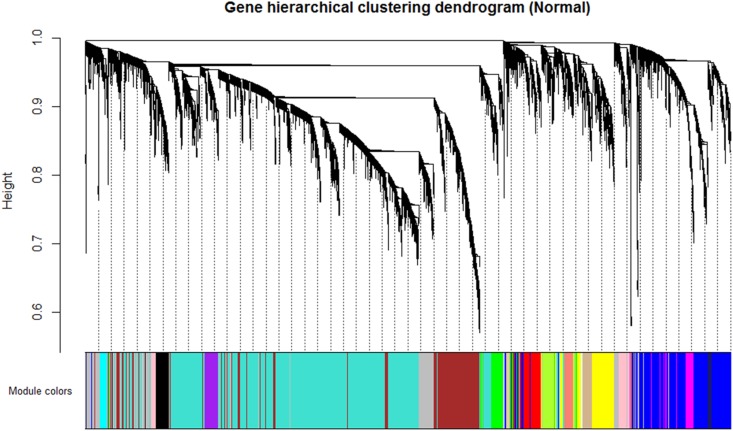
Identification of gene co-expression modules in normal endometrium data (reference data) using average hierarchical linkage clustering; the *y*-axis denotes the co-expression distance and the *x*-axis corresponds to genes. Dynamic tree cutting was applied to identify modules by dividing the dendrogram at significant branch points. Modules are displayed with different colors in the horizontal bar immediately below the dendrogram, with gray representing unassigned genes.

By using the steps described in the Section “Materials and Method,” a network was generated using the genes expressed in NEM and then, DEGs with similar expression pattern were grouped into modules. A total of 16 co-expression modules was identified via hierarchical clustering, which were displayed by different colors according to the WGCNA package function. Resulting gene dendrogram and respective module colors are shown in **Figure 3**. The number of genes per module (module size) ranged from 44 (lightcyan) to 3,858 (turquoise) genes with an average size of 571 genes. Moreover, there were 991 genes that did not share similar co-expression with the other genes in the network and were assigned to gray module. Therefore, gray module was excluded from further analysis (**Supplementary File S4**).

### Network Preservation Analysis

**FIGURE 4:**
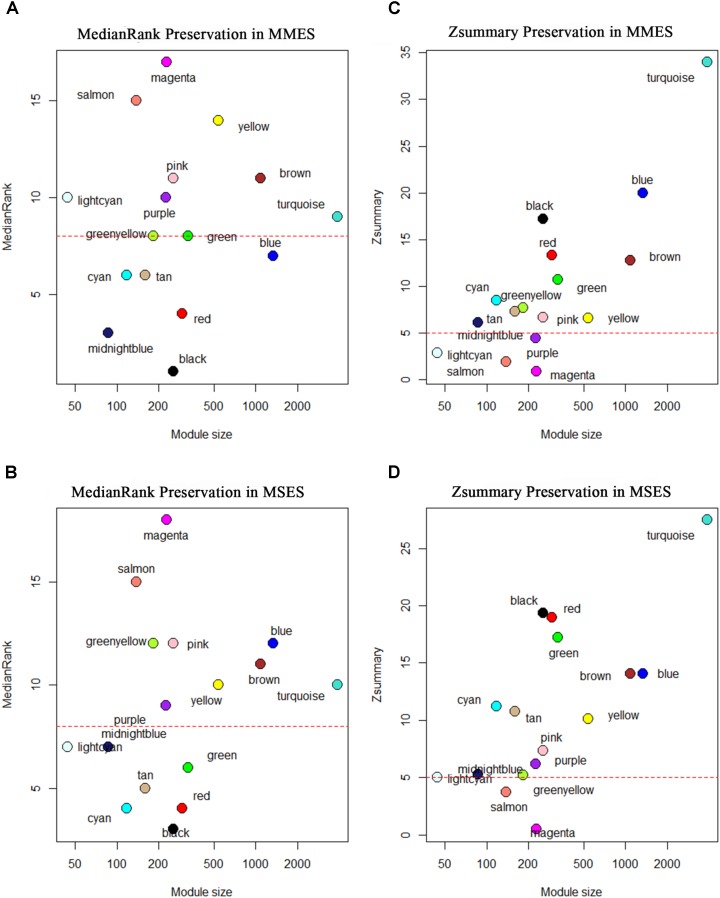
Composite preservation statistics of reference modules after assignment in minimal/mild (MMES) and mild/severe (MSES) ES data (as test data): **(A,B)** the composite statistic *medianRank* (*y*-axis) as a function of the module size; each point represents a module, labeled by color. The dashed red line indicates the threshold *medianRank* = 10; **(C,D)** the summary statistic *Z* (*y*-axis), as a function of the module size; each point represents a module, labeled by color as in **(A)**. The dashed red line indicates the threshold *Z* = 5. A module was considered as non-preserved if it had medianRank ≥ 8 or Zsummary < 5.

After constructing the gene co-expression modules based on the normal samples, we evaluated whether the characteristics of these modules were preserved in the ES stages. Since the connectivity patterns of the non-preserved modules were altered between the normal and ES stages, they may be related to the development of ES. **Figure 4** shows the preservation statistics of modules in MMES and MSES, respectively. Interestingly, all the modules (except two modules, green and blue) had the same preservation patterns in MMES and MSES compared to NEM. Preservation analysis revealed preservation of five modules, including black, cyan, midnight blue, red and tan, between the NEM and ES stages (MMES and MSES) with Zsummary > 5 *and* medianRank ≤ 8. The green module showed preservation in MSES but not in MMES whereas the blue module showed preservation in MMES but not in MSES.

### Non-preserved Modules and Functional Enrichment Analysis

In order to understand the potential molecular mechanisms responsible for NEM to ES transition, we focused on non-preserved modules. According to the determined thresholds (Zsummary < 5 *or* medianRank ≥ 8), nine modules were non-preserved in both MMES and MSES including brown, greenyellow, lightcyan, magenta, pink, purple, salmon, turquoise, and yellow modules. Non-preservation suggests that the expression patterns and network characteristics of the genes in these modules vary across NEM and ES. Therefore, the results indicated that gene co-regulatory patterns in NEM were disrupted by the ES disease. All genes of each module and their module membership values are represented in **Supplementary File S4**.

**FIGURE 5:**
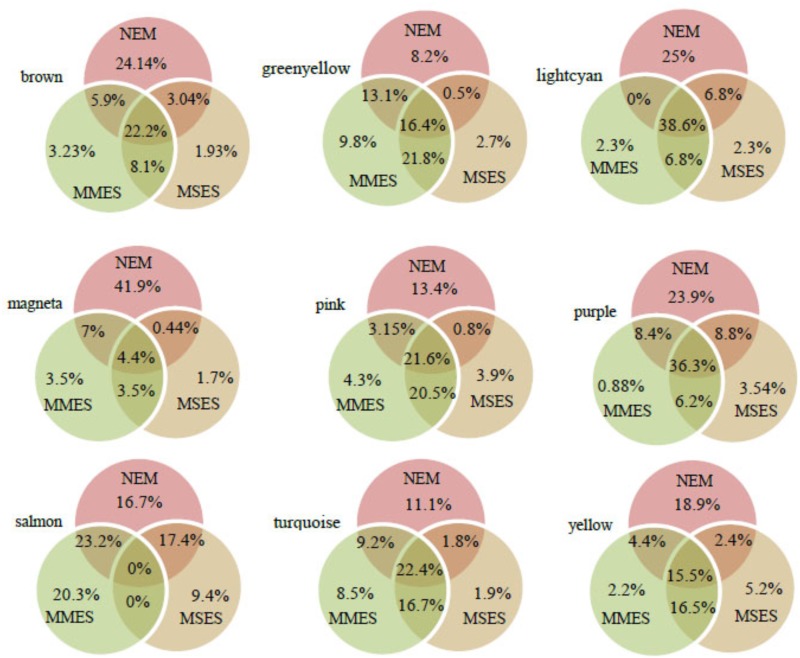
The percentage of hub genes in normal endometrium (NEM), minimal/mild endometriosis (MMES), and mild/severe endometriosis (MSES) in each non-preserved module. The percentages of specific hub genes for each condition, and common hub genes between them were calculated. The total number of hub genes and other details for each module can be found in **Supplementary File S5**.

After assessing the global properties of the non-preserved modules, we investigated the details of these modules. Since hub genes have the highest degree of within-module connectivity, they may play important roles in ES. The potential of hub genes as possible disease associated markers has been reported previously (Horvath, 2011). Therefore, in order to identify hub genes that well represent the non-preserved modules, we analyzed some of these modules in further detail. Here, hub genes were identified in the non-preserved modules by relying on *k*_ME_ values. Genes with *k*_ME_ ≥ 0.7 were considered as hub genes in each module (**Supplementary File S4**). According to **Supplementary File S5**, the highest number of hub genes was found in turquoise, brown and yellow modules, respectively. The lightcyan had the least number of hub genes. However, when the number of specific hubs were expressed in percentage for each module, it was found that magenta and salmon modules contained the highest percentages of specific hubs (**Figure 5**).

In the turquoise module, 1750, 2184, and 1652 hub genes were identified in NEM, MMES, and MSES, respectively (**Supplementary File S4**). In NEM, 427 genes within the turquoise module showed *k*_ME_ ≥ 0.7, while these genes had *k*_ME_ < 0.7 in MMES and MSES. Thus, these genes were identified as specific hub genes of NEM (**Supplementary File S4**). This result implies that the disease disturbed the centrality of these genes. Moreover, 330 and 73 specific hub genes were found in MMES and MSES, respectively (**Supplementary File S5**). Module membership analysis identified 354 common hub genes between NEM and MMES. Further, 863 genes were predicted as common hubs in NEM, MMES, and MSES. Additionally, 70 common hub genes were found between NEM and MSES while 643 common hub genes were found between MMES and MSES. Compared to its large module size (3858 members), the turquoise had a small number of specific and common hub genes in NEM, MMES and MSES. GO analysis of turquoise members showed interesting results. GO terms such as non-coding RNA (ncRNA) metabolic process, gene transcription and ncRNA processing were among the statistically overrepresented GO terms (**Supplementary File S6** and **Table 1**).

**Table 1:** Some of the enriched GO terms in non-preserved modules.

Module	Significant GO terms
Turquoise	ncRNA metabolic process; gene expression; ncRNA processing; translation; tRNA metabolic process; RNA splicing;
Brown	Mitotic cell cycle; nuclear division; cell cycle phase transition; organelle fission; DNA repair; chromosome segregation
Yellow	Response to alcohol; response to steroid hormone; response to reactive oxygen species; cellular response to transforming growth factor beta stimulus; response to transforming growth factor beta; transforming growth factor beta receptor signaling pathway; regulation of inflammatory response; JAK-STAT cascade involved in growth hormone signaling pathway; regulation of response to cytokine stimulus
Magenta	Mitotic nuclear envelope disassembly; membrane disassembly; nuclear envelope disassembly; positive regulation of neuron projection development; regulation of synapse structural plasticity
Lightcyan	Leukocyte cell-cell adhesion; negative regulation of adaptive immune response; positive regulation of cell migration; response to interleukin-15; cellular response to interleukin-4; negative regulation of cytokine production involved in immune response
Pink	mRNA processing
Purple	Actin filament-based process; activation of immune response; regulation of I-kappaB kinase/NF-kappaB signaling; extracellular matrix organization; extracellular structure organization; regulation of cytokine secretion
Greenyellow	Regulation of leukocyte differentiation; antigen processing and presentation of exogenous peptide antigen via MHC class I, TAP-independent; positive regulation of T cell mediated cytotoxicity; cellular response to interferon-gamma; CD4-positive, alpha-beta T cell differentiation; cytokine-mediated signaling pathway; regulation of cytokine production; positive regulation of cytokine production
Blue	Extracellular matrix organization; extracellular structure organization; positive regulation of kinase activity; regulation of neurotransmitter secretion; activation of JUN kinase activity
Green	ncRNA metabolic process; mitochondrial electron transport, NADH to ubiquinone; respiratory electron transport chain; ncRNA processing


In the brown module, 600, 428, and 383 hub genes were identified in NEM, MMES, and MSES, respectively (**Supplementary File S4**). Moreover, 262 specific hub genes were found in NEM (**Supplementary File S5**). Also, 35 and 21 hub genes were specific to MMES and MSES, respectively. Furthermore, 241 hub genes were common between NEM, MMES, and MSES whereas 88 hub genes were common between MMES and MSES. In addition, 64 genes were common hub genes between NEM and MMES while 33 genes were common hubs between NEM and MSES. Compared to the size of the brown module (1085 genes), a lot of these genes were found as hub genes in NEM, MMES, and MSES. This feature is comparable to the turquoise module (**Supplementary File S5**). Most of the significantly overrepresented GO terms were related to cell cycles such as mitotic cell cycle, nuclear division and cell cycle phase transition (**Table 1** and **Supplementary File S6**).

In the yellow module, membership analysis led to the identification of 223, 209, and 214 hub genes in NEM, MMES, and MSES, respectively (**Supplementary File S3**). Moreover, 102, 12, and 28 specific hub genes were found in NEM, MMES, and MSES, respectively (**Supplementary File S5**). Also, 24 hub genes were found between NEM and MMES. In addition, 13 common hubs were found between NEM and MMES while 89 common hub genes were detected between MMES and MSES. Further, 84 genes were common hubs in NEM, MMES, and MSES (**Supplementary File S5**). GO analysis showed that the hub genes of the yellow module were enriched in cellular response to hormone stimulus, response to steroid hormone, cellular response to transforming growth factor beta (TGF-β) stimulus, TGF-β receptor signaling pathway, apoptotic signaling pathway, regulation of inflammatory response and regulation of response to cytokine stimulus (**Table 1** and **Supplementary File S6**).

Gene ontology and pathway enrichment analyses of the other non-preserved modules were performed to assess whether they were significantly enriched for genes belonging to specific GO terms or pathways (**Table 1** and **Supplementary File S6**). The only salmon module did not have significant GO term. Genes in the lightcyan as the smallest module were enriched in negative regulation of adaptive immune response, positive regulation of apoptotic signaling pathway and response to interleukins. In the magenta module, GO terms related to the nerve system were overrepresented such as positive regulation of dendrite development, regulation of synapse structural plasticity and positive regulation of neuron projection development. Genes in the purple module were enriched in I-kappaB kinase/NF-kappaB signaling regulation, extracellular matrix organization, immune response activation and immune response-activating signal transduction.

We further hypothesized that hub genes within a non-preserved module were co-regulated by common TFs. To test this hypothesis, the hub genes in each module were analyzed. The results showed that all the groups had at least one significant TFBS except the salmon, pink and lightcyan modules, which indicated that the hub genes in each non-preserved module were co-regulated. Although the turquoise and brown modules had a large number of hub genes, only 12 and 2 common TFBSs were found at promoters of their hub genes, respectively. The highest number of common TFBSs was found at promoters of the hub genes of the yellow module (35 TFBSs). The E2F1 binding site was detected at promoters of the hub genes of the brown module. The MYB, GATA6, and ATF4 binding sites were detected only in the turquoise module. The SMAD4 binding site was detected at promoters of the hub genes of both the turquoise and purple modules. The ZNF148, KLF11, and KLF4 binding sites were found at promoters of the hub genes of the yellow and greenyellow modules. Further, the NFKB1, TEAD4, and TEAD2 binding sites were only found at promoters of the hub genes of the yellow module. All the results of this section are presented in **Supplementary File S6**.

### Preserved Modules and Functional Enrichment Analysis

Functional enrichment analysis showed that all the preserved modules except the midnight blue were significantly enriched with genes in different significant GO terms and KEGG pathways, which provided evidence of functional role for each module as a whole. Some of the most highly significantly GO terms and KEGG pathways in each of the preserved modules were as follows: endosomal transport and regulation of translation (black), proline metabolic process and regulation of translation (cyan), metabolic pathways and glycosaminoglycan biosynthesis (red), GTP metabolic process (tan), alanine, aspartate and glutamate metabolism (tan). In addition, the analysis of the conserved TFBSs revealed that 45 significant TFBSs were over-represented in all the preserved modules except the tan module ranging from one (the black module) to 32 (the red module) TFBSs. These findings indicated that signed WGCNA could effectively classify co-regulated and biologically related genes into separate modules. The complete results of the functional enrichment and promoter analysis for the preserved modules are presented in **Supplementary File S7**.

### Validation by Real-Time RT-PCR

**FIGURE 6:**
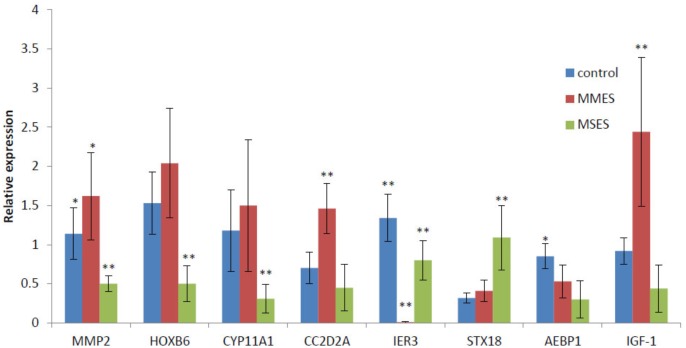
The results of RT-PCR analysis of eight gene candidates in NEM, MMES, and MSES; ^∗^ and ^∗∗^ means are significantly different at 0.05 and 0.01 probability level, respectively.

To learn whether the expression patterns of the hub genes could be recapitulated using other independent samples, we selected eight genes which gained or lost their hubness in NEM, MMES, or MSES, and checked their expression in healthy women and women with MMES and MSES using real-time RT-PCR. Five new genes including CC2D2A, AEBP1, HOXB6, IER3, and STX18 along with three previously known genes including MMP-2, CYP11A1, and IGF-1 were selected for the test. Interestingly, the expression analysis showed significant changes in expression level of the genes in different stages (**Figure 6**). For example, IER3 gained *k*_ME_ more than 0.7 in the MMES and MSES stages (0.8 and 0.77, respectively, and 0.54 in NEM). In accordance with the results, expression of IER3 strongly decreased in the MMES stage, but then increased in the MSES stage. Note that IER3 was a hub gene in MMES due to its dramatic downregulation. This may suggest important role for this gene during the development of the disease. Among the genes, we selected some previously known genes such as MMP-2. In our analysis, MMP-2 was defined as a hub gene in both NEM and MMES. Its expression also increased from the NEM to MMES stage. Although its expression decreased in MSES, it seemed that MMP-2 was highly affected in MMES rather than in MSES.

CYP11A1 was also a hub gene in MSES but not in MMES, and according to our results, its expression dramatically decreased in MSES. This may suggest that CYP11A1 has important role in progress of the disease into the MSES stage. CC2D2A was upregulated in MMES and then, decreased in MSES to its level of expression in NEM. In accordance with our results, it was a hub gene in MMES and MSES. More interestingly, STX18 showed a small increase in MMES and then, an increase in the MSES stage compared to NEM. It was defined as a hub gene in MMES and MSES. AEBP1 gradually decreased from NEM to MSES but obtained a high score in MSES, highlighting its important role in the MSES stage. HOXB6 increased in MMES and decreased in MSES, but according to the results, it was a hub gene in MMES. To our knowledge, this is the first report of CC2D2A, AEBP1, HOXB6, IER3, and STX18 in different stages of ES. The same result was found for IGF-1, which showed a high expression in MMES compared to NEM and MSES. This gene was a hub gene in MMES.

## Discussion

Our analysis revealed insights into the molecular mechanisms underlying the ES disease that are consistent with previously published studies. Still, the detailed network analyses provided an increased understanding of the role of specific and common key regulators associated with NEM and ES stages. Weighted gene co-expression networks enabled us to predict group(s) of genes whose expressions were highly correlated in a specific tissue/condition. In these networks, highly connected nodes can represent essential genes that may contribute to a disease or phenotype.

In contrast to focusing on DEGs, the construction of gene co-expression modules led us to the identification of candidate genes that were highly associated with the disease. Differentially expressed genes in NEM were selected and a number of modules were constructed using WGCNA. These modules were tested with data from MMES and MSES patients to investigate whether they are preserved in these two disease states. If the modules were not preserved, we looked for the hub genes which responsible for the perturbation of the network. In WGCNA, there are two types of the connectivity measure: one measure is based on the connectivity of a gene in respect to whole network, and the other measures the connectivity of a gene with respect to the other genes within a particular module which is called intra-modular connectivity. Previous studies showed that the second measure is more effective for selection of biologically important hub genes (Ghazalpour et al., 2006). Therefore, this method has been used to identify the respective hub genes in the different disease states.

Our results implied that the normal expression and connectivity of the genes within the modules were disturbed by the ES disease. One important finding was that the preservation pattern of the 14 modules was similar between MMES vs. NEM and MSES vs. NEM. The consistency of the module preservation in both comparisons indicated the robustness and reproducibility of the changes in the gene co-expression patterns. Genetic susceptibility, estrogen dependence, progesterone resistance and inflammation are the hallmarks of the ES disease and in our study, GO terms related to these biological processes were significantly overrepresented in the non-preserved modules, but not in the preserved modules (**Table 1**). Interestingly, two modules (green and blue) were also not preserved between MMES and MSES. Little is known about different molecular mechanisms between MMES and MSES. The gene members of these two modules can yield important information about the progression of the disease from mild to severe stage.

Previous studies have identified several genes involved in the ES disease (**Table 2**). These genes were also predicted by WGCNA in this study. Furthermore, we could identify genes that are co-expressed with these candidate genes and predict, if these genes are in the same or different modules. Three genes, MMP-2, CYP11A1, and IGF-1, were validated in our analysis. MMP-2, MMP-16, and MMP-19 are matrix metallopeptidases, where MMP-16, and MMP-19 are predicted as hub genes only in NEM in the different modules, MMP-2 was predicted as hub gene in both NEM and MMES. In accordance with our results, higher MMP-2 level was found in sera and peritoneal fluids of patients with ES (Huang et al., 2004). It was demonstrated that MMP-2 concentration was associated with steroid hormones in these patients and its expression increased in eutopic endometrium from patients with ES (Chung et al., 2002). CYP11A1 is a steroidogenic gene and is highly expressed in endometriotic tissue. (Attar et al., 2009). Another member of the cytochrome 540 family, CYP19A1, was identified in a module(blue), which differed between MSES and MMES. It has been previously reported that levels of IGF-I in the peritoneal fluid are significantly higher in patients with ES compared with those in normal women (Kim et al., 2000).

**Table 2:** A number of previously identified genes which were predicted in non-preserved modules; most of the genes built hub in the modules.

Gene name	Hubness	Found in module	Reference
TGF-β1	Hub	Yellow	Oosterlynck et al., 1994
ILs	No hub	Brown, turquoise	Andreoli et al., 2011
BCL-2	Hub	Yellow	Jones et al., 1998
17β-HSD	Hub	Turquoise	Zeitoun et al., 1998
PGR	Hub	Brown	Attia et al., 2000
ESR1	Hub	Brown	Jeong et al., 2005
CLDNs	Hub	Yellow, greenyellow, turquoise	Jeong et al., 2005
HOXA10	Hub	Brown	Taylor et al., 1999
COL1A1	Hub	Magenta	Hull et al., 2008
PTEN	Hub	Brown	Sato et al., 2000
MMP-2	Hub	Greenyellow	Huang et al., 2004


Moreover, the inflammatory condition of ES has been reported previously. In our results, we also found traces of genes involved in inflammation such as interleukins (IL) and TGF-β1. TGF-β1 was a hub gene in all NEM, MMES, and MSES networks in t in one module (yellow). A previous study demonstrated that TGF-β1 activity was highly elevated in peritoneal fluid in women with ES (Oosterlynck et al., 1994). Other interleukins, namely IL7, IL18, and IL32, were found in the turquoise and brown modules but were not defined as hub genes. Instead, some IL receptors were found as hub genes such as IL17RB (in NEM), IL13RA1 (in both MMES and MSES), IL12RB1 (in NEM and MMES) and IL2RB1 (in NEM, MMES, and MSES). IL10RB was also a member of the turquoise module. The levels of IL-10, IL-12, and IL-17 proteins were comparable between infertile controls and ES patients with infertility (Andreoli et al., 2011). These findings highlight the importance of IL signaling for the disease.

A member of the B-cell lymphoma/leukaemia2 genes (BCL-2), BCL2L1, was found in the yellow modules a hub gene in MMES and MSES but not in NEM. The B-cell lymphoma/leukaemia2 genes are anti-apoptotic and proto-oncogene. It was also reported that BCL-2 was upregulated in eutopic and ectopic ES compared to NEM (Jones et al., 1998). In addition, apoptotic cells were rarely found in ES (either eutopic or ectopic endometrium) and NEM (Jones et al., 1998). These results are in line with our finding and demonstrate the central role of BCL-2 in ES.

Endometriosis is an estrogen dependent disease and its molecular evidence is the low expression of 17β-HSD in ES vs. NEM (Zeitoun et al., 1998). Interestingly, 17β-HSD was one of the hub genes in our analysis. 17β-HSD inactivates estradiol to estrone and is expressed in the luteal eutopic endometrium in response to progesterone but not in simultaneously biopsied peritoneal endometriotic tissue (Attia et al., 2000). Progesterone receptor (PGR or PR) was found as a hub gene in NEM, MMES, and MSES. Attia et al. (2000) reported that the total level of PGR was reduced in ES in contrast to eutopic endometrium. In addition, estrogen receptor α (*ESR1*) and Claudins (*CLDNs*) as progesterone-responsive genes (Jeong et al., 2005) were identified as hub genes in ES rather than in NEM. In the healthy endometrium, both ESR1 and ESR2 are expressed, although ESR1 predominates over ESR2, and their expression differs during the menstrual cycle (Matsuzaki et al., 2001). Estrogen-mediated proliferation in endometrium is promoted mainly through the activation of ESR1 (Shang, 2006). Previous studies have shown down-regulation of ESR1 in different types of endometriosis lesions (Matsuzaki et al., 2001).

*HOXA10* is a homeobox gene (estrogen progesterone responsive gene), the expression of which alters in response to sex steroids during the menstrual cycle, with dramatic up-regulation in the mid-secretory phase, the time of implantation. However, it is dramatically downregulated in patients with ES (Taylor et al., 1999). Interestingly, in our analysis, this gene was a specific hub gene in MMES. HOXA10 regulates expression of various downstream genes including those encoding cell adhesion molecules, signal transduction factors, transcription factors, and metabolic mediators (Zanatta et al., 2010).

Because, a GO term related to ncRNAs was significantly enriched in the turquoise module we were interested to compare all members of the non-preserved modules with miRNA targets in ES identified by Ohlsson Teague et al. (2009). This study identified 22 differentially expressed miRNAs and their predicted targets (3851 different mRNAs) in ES (Ohlsson Teague et al., 2009). Interestingly, as shown in **Table 3**, 535 genes in the turquoise module were predicted as the targets of differentially expressed miRNAs in ES. This module had the highest number of target genes compared to other modules and as shown by GO analysis, ncRNA metabolic process was the most significant GO term in the turquoise module. Moreover, 49 of these target genes were found as specific hubs in NEM in the turquoise module. Based on our WGCNA analysis, the hubness and centrality of these genes were lost by the ES disease in either MMES or MSES stages. These results nicely show the power of network analyses with WGCNA. The list of miRNA target genes found in each module is represented in **Supplementary File S8**.

**Table 3:** Number of miRNA target genes found in non-preserved modules; all members of each module were compared with 3851 target genes predicted as the targets of 22 differentially expressed miRNAs in ES tissue by Ohlsson Teague et al. (2009).

Module name	Turquoise	Brown	Yellow	Greenyellow	Lightcyan	Pink	Magenta	Purple	Salmon
Number of miRNA targets	535 (13.87%)	167 (15.4%)	91 (16.85%)	22 (12.02%)	7 (15.91%)	36 (14.17%)	28 (12.23%)	48 (21.24%)	17 (12.32%)


Collagen, COL1A1, was another specific hub in NEM; but not in MMES and MSES. It is demonstrated that transcription factor KLF11 recruits SIN3A to repress COL1A1 and therefore, its inhibition results in increased fibrosis (Zheng et al., 2016). A previous study also reported that the expression of COL1A1 increased in human ectopic endometrial lesions (Hull et al., 2008). It is worth noting that KLF11 and SIN3A were members of the turquoise module in this study and their expressions significantly decreased in MMES and MSES vs. NEM. Also, COL1A1 as a member of the magenta module was significantly over-expressed in MMES and MSES vs. NEM. We detected significant over-representation of the binding site of KLF11 on magenta module genes (*p*-value = 0.01).

K-RAS was another hub in NEM in the turquoise module. K-RAS is an oncogene and its mutation induces ES (Dinulescu et al., 2005). In our WGCNA analysis, K-RAS lost its hubness in MMES. Endometrium is vulnerable to errors of genetic recombination (Wu et al., 2006). PTEN, a tumor suppressor gene, is an evidence for genomic alteration in ES. Loss of heterozygosity and frequent somatic mutations in *PTEN* were found in 56 and 21% of solitary endometrial cysts of the ovary, respectively (Sato et al., 2000). Importantly, in our results, PTEN was a common hub in MMES and MSES but not in NEM. Moreover, a mutation in both *K-RAS* and *PTEN* leads to invasive and metastatic ovarian cancer (Dinulescu et al., 2005). Thus, it is suggested that WGCNA can help to find common or discrete pathways between ES and ovarian cancer.

We also validated the expression of several new genes in ES, including CC2D2A, AEBP1, HOXB6, IER3, and STX18. CC2D2A is a Meckel syndrome gene and its mutation causes embryonic lethality (Tallila et al., 2008). AEBP1 is potent modulator of NF-kappaB and involved in inflammatory processes (Majdalawieh and Ro, 2010). HOXB6 is a homo-box DNA binding protein and predicted as a miRNA target in ovarian ES (Filigheddu et al., 2010). IER3 is a stress-inducible protein and regulates cell proliferation and apoptosis. IER3 expression is downregulated in ovarian carcinoma (Han et al., 2011). STX18 is a target-soluble *N*-ethylmaleimide-sensitive factor-attachment protein receptor and may function in transport between endoplasmic reticulum and Golgi apparatus (Harada et al., 1997).

To summarize these findings, many candidate genes where found to be important for different disease states. Although many of these genes have been described previously in relation to ES, our analysis reveals novel networks and a specificity of mild and severe disease states.

## Conclusion

In this study, the comparison between memberships of the genes in different modules demonstrated that many previously studied genes were present within the non-preserved modules. The relationships of these genes with each other or with other unknown genes were not identified previously. As discussed, some of these genes were found in different modules. Many known genes in the non-preserved modules changed their position and centrality in the different networks between NEM or ES. Apart from confirming known genes, many new genes that have not been studied previously have been identified in different modules. Our use of signed WGCNA shed light on the importance of unknown genes and their co-expressed partners. These genes may play significant roles in the pathophysiology of ES. For example, nuclear import of NF-κB essential modulator (NEMO) is necessary for DNA damage-dependent NF-κB signaling. Importins (IPOs) promote NEMO’s nuclear import (Hwang et al., 2015). In the brown module, IPO9 could build a common hub in MMES and MSES. Another interesting gene was protein tyrosine phosphatase localized to the Mitochondrion 1 (PTPMT1), which was a specific hub in MMES in the turquoise module. PTPMT1 is localized in mitochondria and its downregulation is sufficient to promote cancer cell death (Mukund and Subramaniam, 2015). Although ES and cancer are similar in some aspects such as development of new blood vessels and decrease in the number of cells undergoing apoptosis, ES is not a malignant disorder (Swiersz, 2002). The results of the present study could help to better understand similarities and differences between ES and cancer as well as the differences in the progression of ES.

Prostaglandin D_2_ synthase (PTGDS) was also a specific hub in NEM in the salmon module, which was downregulated in NEM compared to ES. PTDGS is stimulated by estrogen and catalyzes prostaglandin H_2_ to prostaglandin D_2_ that exhibits functions including regulation of the central nervous system, contraction/relaxation of smooth muscle and inhibition of platelet aggregation (Lim et al., 2015).

We used available microarray datasets for WGCNA. But our framework easily handles high throughput sequencing data as well. The incorporation of these data will further extend the gene co-expression network. In contrast to microarray technique, next generation sequencing can identify a number of new differentially expressed genes, thus incorporating such data might help to extend small modules or to break down the large modules into distinct smaller modules. Considering the disease is complicated and affected by a number of internal and external factors such as the physiological status of the patients, age, the time of sampling, population structure and etc., deep sequencing with high number of biological replications is recommended. Further expression analysis at protein level or functional analysis of the genes is suggested in the future to incorporate this system-level understanding of the gene into the practice of building effective prognostic models.

## Author Contributions

MRB analyzed the data. MS carried out RT-PCR experiment. AK and PG contributed to the interpretation and critically revised the manuscript. BH interpreted the results, wrote the manuscript, and conceived and designed the study.

## Conflict of Interest Statement

PG is employed by company AI VIVO Ltd. The remaining authors declare that the research was conducted in the absence of any commercial or financial relationships that could be construed as a potential conflict of interest. The reviewers MM-S and AM and the handling Editor declared their shared affiliation.

## Supplementary Material

FIGURE S1 Scale free topology plot for choosing the power β for the signed correlation network; left-hand side: the SFT index *R*^2^ (*y*-axis) as a function of different powers β (*x*-axis). The first β is used where the saturation curve is seen as long as *R*^2^ is above 0.8; right-hand side: the mean connectivity (*y*-axis) is a strictly decreasing function of the power β (*x*-axis).

FILE S1 R script used for the analysis in this study.

FILE S2 List of the primers used for RT-PCR experiments.

FILE S3 Differentially expressed genes in normal endometrium and ES disease at minimal/mild and mild/severe stages based on Tamaresis et al. (2014)

FILE S4 Properties of co-expression modules using signed WGCNA in normal endometrium data generated by Tamaresis et al. (2014) study; module membership or intramodular connectivity measures (kIM and kME) were calculated for non-preserved modules.

FILE S5 List of specific hub genes of NEM, MMES, and MSES, and common hub genes between them in each non-preserved module.

FILE S6 Gene ontology and KEGG pathway enrichment analyses of identified non-preserved modules and prediction of common TF binding sites on their hub genes.

FILE S7 Gene ontology and KEGG pathway enrichment analyses of identified preserved modules and prediction of common TF binding sites on their hub genes.

FILE S8 List of target genes of ES differentially expressed miRNAs predicted by Ohlsson Teague et al. (2009) that were found in each non-preserved module.
