# Supplementary figures and images for: Weighted Gene Co-expression Network Analysis of Endometriosis and Identification of Functional Modules Associated With Its Main Hallmarks
Source: Front Genet. 2018 Oct 12;9:453. doi: 10.3389/fgene.2018.00453 (PMC6194152; doi:10.3389/fgene.2018.00453)

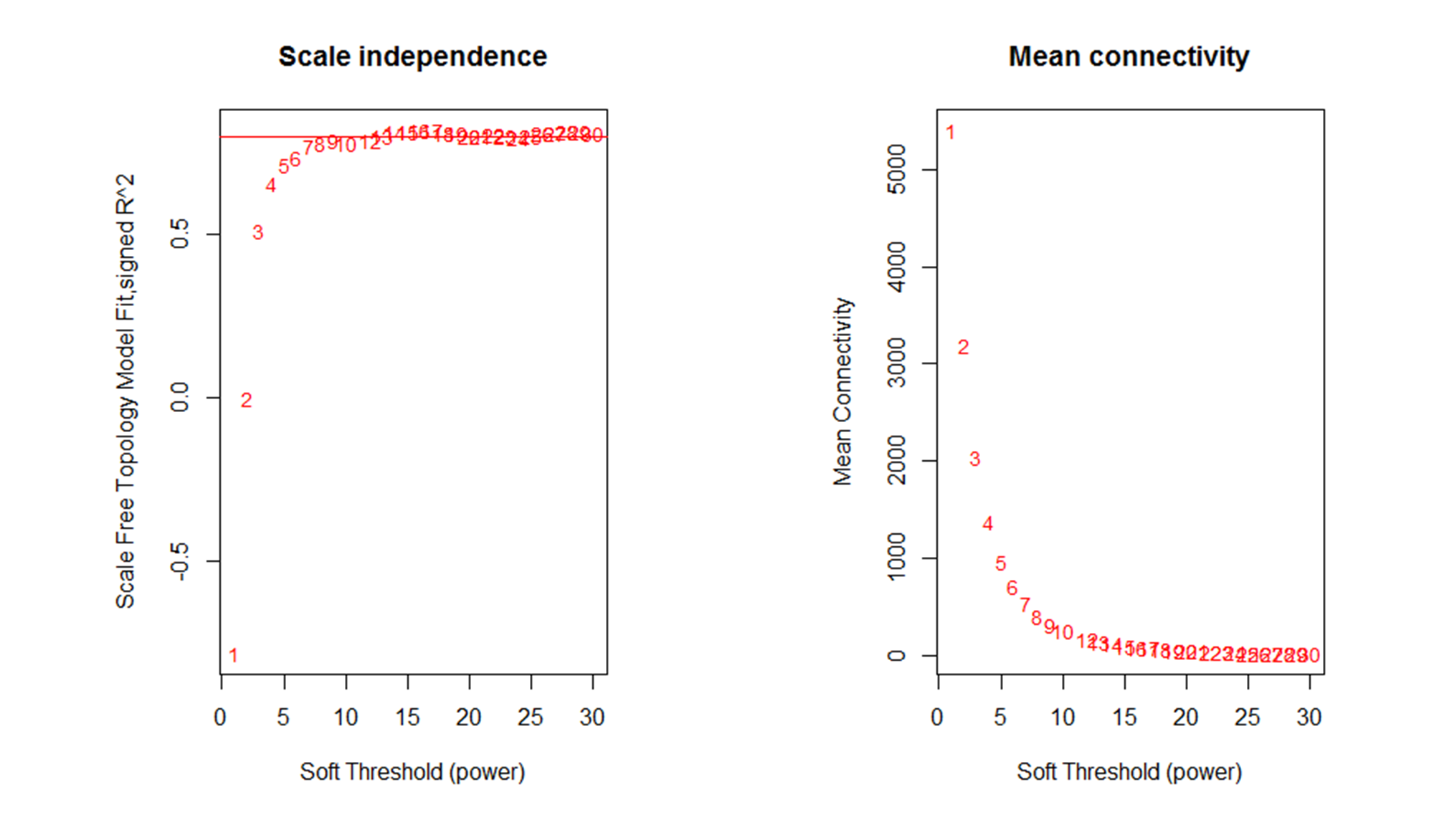

Supplement: FIGURE S1 — Scale free topology plot for choosing the power β for the signed correlation network; left-hand side: the SFT index R2 (y-axis) as a function of different powers β (x-axis). The first β is used where the saturation curve is seen as long as R2 is above 0.8; right-hand side: the mean connectivity (y-axis) is a strictly decreasing function of the power β (x-axis). [file Image_1.TIF]
