# Supplementary material for: Weighted Gene Co-expression Network Analysis of Endometriosis and Identification of Functional Modules Associated With Its Main Hallmarks
Source: Front Genet. 2018 Oct 12;9:453. doi: 10.3389/fgene.2018.00453 (PMC6194152; doi:10.3389/fgene.2018.00453)
Supplement: FILE S1 — R script used for the analysis in this study. [file Data_Sheet_1.pdf]

```
rm(list=ls())


####################
rm(list=ls())
load(file ="0-gset_GEO.RData")
load(file ="0-NoSe.RData")
load(file ="0-NoMi.RData")

# The negative Fc = expression is higher in Normal, Threshod=1.5
Filter_NS  <- row.names(subset(NoSe, abs(logFC) > 0.18 &  adj.P.Val   <
0.05))

Filter_NM  <- row.names(subset(NoMi, abs(logFC) > 0.18 &  adj.P.Val   <
0.05))

shared =as.data.frame(merge(Filter_NM,Filter_NS,by=1,sort=F)[,1])      #
Or shared=intersect(Filter_NM,Filter_NS)
row.names(shared) <- shared[,1]

expression= as.data.frame(exprs(gset))

myData_Normalized_Expr_Filt =merge(expression,shared,by = "row.names",
all = F)
row.names(myData_Normalized_Expr_Filt)=myData_Normalized_Expr_Filt[,1]
rem <- c("Row.names","merge(Filter_NM, Filter_NS, by = 1, sort = F)[,
1]")
myData_Normalized_Expr_Filt = myData_Normalized_Expr_Filt[ ,
!(names(myData_Normalized_Expr_Filt) %in% rem)]
dim(myData_Normalized_Expr_Filt)

####################



#################### Convert Affymetrix ID to gene SYMBOL or ENTREZID or
GENENAME. select(hgu133plus2.db, PROBES, c("SYMBOL", "ENTREZID",
"GENENAME"))
library("hgu133plus2.db")

SYMBOL <- data.frame(SYMBOL=sapply(contents(hgu133plus2SYMBOL), paste,
collapse=", ") )

myData_Normalized_Expr_Filt_Symbol <- merge(SYMBOL,
myData_Normalized_Expr_Filt, by.x=0, by.y=0, all.y=T)
row.names(myData_Normalized_Expr_Filt_Symbol) <-
myData_Normalized_Expr_Filt_Symbol[,1]
myData_Normalized_Expr_Filt_Symbol <-
myData_Normalized_Expr_Filt_Symbol[,-1]

####################
```

```
#################### Averge genes with several probset by collapseRows of
WGCNA
library(WGCNA)

datET <- myData_Normalized_Expr_Filt_Symbol[,-1]
rowGroup <- myData_Normalized_Expr_Filt_Symbol[,1]
rowID <- rownames(datET)

collapse.object=collapseRows(datET=datET, rowGroup=rowGroup,
rowID=rowID,method="MaxMean")    #  method="maxRowVariance"   or MaxMean

myData_Normalized_Expr_Filt_Symbol_Coll=data.frame(
collapse.object$group2row, collapse.object$datETcollapsed)
myData_Normalized_Expr_Filt_Symbol_Coll=myData_Normalized_Expr_Filt_Symbo
l_Coll[,-c(1,2)]

####################



########################## WGCNA
allowWGCNAThreads()
suppressMessages(library(cluster))
options(stringsAsFactors = FALSE);

drops=c("GSM1256795","GSM1256794","GSM1256792","GSM1256790","GSM1256789",
"GSM1256783","GSM1256765","GSM1256764","GSM1256763","GSM1256762","GSM1256
761","GSM1256760","GSM1256759","GSM1256758","GSM1256757","GSM1256756","GS
M1256755","GSM1256754","GSM1256753","GSM1256752","GSM1256751","GSM1256750
","GSM1256749","GSM1256748","GSM1256747","GSM1256746","GSM1256745","GSM12
56744","GSM1256743","GSM1256742","GSM1256741","GSM1256740","GSM1256739","
GSM1256738","GSM1256737","GSM1256736","GSM1256735.CEL","GSM1256696","GSM1
256702")
df =
myData_Normalized_Expr_Filt_Symbol_Coll[,!(names(myData_Normalized_Expr_F
ilt_Symbol_Coll) %in% drops)]
datExpr = as.data.frame(t(df))


########################## Outlier detection
A = adjacency(t(datExpr), type = "distance")
k = as.numeric(apply(A, 2, sum)) - 1
Z.k = scale(k)
thresholdZ.k = -2.5  # often -2.5
outlierColor = ifelse(Z.k < thresholdZ.k, "red", "black")
sampleTree = hclust(as.dist(1 - A), method = "average")
datColors = data.frame(outlierC = outlierColor)



############## Remove outlying samples from expression  data
remove.samples = Z.k < thresholdZ.k | is.na(Z.k)
datExpr = datExpr[!remove.samples, ]
```

```
A = adjacency(t(datExpr), type = "distance")
k = as.numeric(apply(A, 2, sum)) - 1
Z.k = scale(k)
##############
##########################




########################## Divide disease and normal samples
load(file ="4-datExpr_RemSam.RData")

# Remove "GSM1256685"  "GSM1256686"
datExpr_m =
as.data.frame(datExpr[c("GSM1256778","GSM1256776","GSM1256775","GSM125677
4","GSM1256718","GSM1256715","GSM1256713","GSM1256708","GSM1256706","GSM1
256705","GSM1256700","GSM1256698","GSM1256694","GSM1256693","GSM1256692",
"GSM1256691","GSM1256690","GSM1256689","GSM1256688","GSM1256684","GSM1256
683","GSM1256682","GSM1256677","GSM1256674","GSM1256672"),])

# Remove  "GSM1256678" "GSM1256679"
datExpr_s = as.data.frame(datExpr[c(
"GSM1256782","GSM1256781","GSM1256780","GSM1256779","GSM1256777","GSM1256
773","GSM1256719","GSM1256717","GSM1256716","GSM1256714","GSM1256712","GS
M1256711","GSM1256710","GSM1256709","GSM1256707","GSM1256704","GSM1256703
","GSM1256701","GSM1256699","GSM1256697","GSM1256695","GSM1256687","GSM12
56681","GSM1256680","GSM1256676","GSM1256675","GSM1256673","GSM1256671","
GSM1256670","GSM1256669","GSM1256668","GSM1256667","GSM1256666","GSM12566
65","GSM1256664","GSM1256663","GSM1256662","GSM1256661","GSM1256660","GSM
1256659","GSM1256658","GSM1256657","GSM1256656","GSM1256655","GSM1256654"
,"GSM1256653"),])

datExpr_n =
as.data.frame(datExpr[c("GSM1256800","GSM1256799","GSM1256798","GSM125679
7","GSM1256796","GSM1256793","GSM1256791","GSM1256788","GSM1256787","GSM1
256786","GSM1256785","GSM1256784","GSM1256772","GSM1256771","GSM1256770",
"GSM1256769","GSM1256768","GSM1256767","GSM1256766","GSM1256734","GSM1256
733","GSM1256732","GSM1256731","GSM1256730","GSM1256729","GSM1256728","GS
M1256727","GSM1256726","GSM1256725","GSM1256724","GSM1256723","GSM1256722
","GSM1256721","GSM1256720"),])


# Check the matrix

multi=list(Data1=list(data=datExpr_n),
Data2=list(data=datExpr_s),Data3=list(data=datExpr_m))
multi_g=goodSamplesGenesMS(multi)

datExpr_n=datExpr_n[,multi_g$goodGenes]
goodSamplesGenes(datExpr_n)


datExpr_s=datExpr_s[,multi_g$goodGenes]
goodSamplesGenes(datExpr_s)
```

```
datExpr_m=datExpr_m[,multi_g$goodGenes]
goodSamplesGenes(datExpr_m)


############## Choose a set of soft-thresholding powers
load(file ="4-datExpr_n.RData")
load(file ="4-datExpr_s.RData")
load(file ="4-datExpr_m.RData")

library(WGCNA)
allowWGCNAThreads()
suppressMessages(library(cluster))
options(stringsAsFactors = FALSE);


powers = c(c(1:10), seq(from = 12, to=30, by=1))
sft = pickSoftThreshold(datExpr_n, powerVector = powers,
networkType="signed", corFnc = "bicor", verbose = 5,blockSize=17000)   #
corFnc = "bicor"  Or corFnc = cor, corOptions = list(use = 'p')


sizeGrWindow(9, 5)
par(mar=c(6,8,4,4))     #c(bottom, left, top, right)
par(mfrow = c(1,2));
cex1 = 0.9;

plot(sft$fitIndices[,1], -
sign(sft$fitIndices[,3])*sft$fitIndices[,2],xlab="Soft Threshold
(power)",ylab="Scale Free Topology Model Fit,signed R^2",type="n", main =
paste("Scale independence"));
text(sft$fitIndices[,1], -
sign(sft$fitIndices[,3])*sft$fitIndices[,2],labels=powers,cex=cex1,col="r
ed");
abline(h=0.80,col="red")

plot(sft$fitIndices[,1], sft$fitIndices[,5], xlab="Soft Threshold
(power)",ylab="Mean Connectivity", type="n", main = paste("Mean
connectivity"))
text(sft$fitIndices[,1], sft$fitIndices[,5], labels=powers,
cex=cex1,col="red")

##############




############## Comparison of  mean expression level and connectivity
between two data

Data1_mean = as.data.frame((t(datExpr_n)[, -c(1:1)]));
rownames(Data1_mean) = names(datExpr_n);

Data2_mean = as.data.frame((t(datExpr_s)[, -c(1:1)]));
```

```
rownames(Data2_mean) = names(datExpr_s);

Data3_mean = as.data.frame((t(datExpr_m)[, -c(1:1)]));
rownames(Data3_mean) = names(datExpr_m);


par(mfrow=c(1,3))
par(mar=c(6,6,4,4))     #c(bottom, left, top, right)

mean.Data1=apply(Data1_mean,1,mean)
mean.Data2=apply(Data2_mean,1,mean)
mean.Data3=apply(Data3_mean,1,mean)

#plot the result between Data1 and Data2
verboseScatterplot(mean.Data1,mean.Data3,corFnc = "bicor",xlab="Normal",
ylab="Mild", abline =TRUE,abline.color=2,abline.lty =5)
verboseScatterplot(mean.Data1,mean.Data2,corFnc =
"bicor",xlab="Normal",ylab="Severe", abline
=TRUE,abline.color=2,abline.lty =5)
verboseScatterplot(mean.Data3,mean.Data2,corFnc =
"bicor",xlab="Mild",ylab="Severe", abline =TRUE,abline.color=2,abline.lty
=5)

title("Mean expression comparison", outer=TRUE,line = -1)
##############



############## Explores the preservation of connectivity between two data

par(mfrow=c(1,3))
par(mar=c(6,6,4,4))     #c(bottom, left, top, right)

sftData1=softConnectivity(datExpr_n,corFnc = "bicor",type = "signed",
blockSize =17000,minNSamples=5, power=13 )    # nedd power by
pickSoftThreshold
sftData2=softConnectivity(datExpr_s,corFnc = "bicor",type = "signed",
blockSize =17000,minNSamples=5, power=16)
sftData3=softConnectivity(datExpr_m,corFnc = "bicor",type = "signed",
blockSize =17000,minNSamples=5, power=30)

#plot the result between Data1 and Data2
verboseScatterplot(sftData1,sftData3,type = "signed", corFnc = "bicor",
blockSize = 15000, xlab="Normal",  ylab="Mild", abline
=TRUE,abline.color=2,abline.lty =5)
verboseScatterplot(sftData1,sftData2,type = "signed", corFnc =
"bicor",blockSize = 15000, xlab="Normal",ylab="Severe", abline
=TRUE,abline.color=2,abline.lty =5)
verboseScatterplot(sftData3,sftData2,type = "signed", corFnc =
"bicor",blockSize = 15000, xlab="Mild",ylab="Severe", abline
=TRUE,abline.color=2,abline.lty =5)

##############
```

```
############## Module detection. Power and other things have to be set
net1 = blockwiseModules(datExpr_n, power = 13,corType= "bicor",
networkType = "signed",TOMType = "signed", maxBlockSize=17000,
minModuleSize = 30,reassignThreshold = 0, mergeCutHeight = 0.25,
numericLabels = TRUE, pamRespectsDendro = FALSE, saveTOMs = TRUE,
saveTOMFileBase = "5-Data1TOM",nThreads = 6, verbose = 3)
table(net1$colors)



# open a graphics window
sizeGrWindow(12, 9)
# Convert labels to colors for plotting
mergedColors = labels2colors(net1$colors)
# Plot the dendrogram and the module colors underneath
plotDendroAndColors(net1$dendrograms[[1]],
mergedColors[net1$blockGenes[[1]]], "Module colors",  dendroLabels =
FALSE, hang = 0.03, addGuide = TRUE, guideHang = 0.05,
                    main="Gene hierarchical clustering dendrogram
(Normal)" )

##############



############## Save the modules Data1
probes=names(datExpr_n)

moduleLabelsAutomatic1 = net1$colors
moduleColorsAutomatic1 = labels2colors(moduleLabelsAutomatic1)
moduleColorsAutomaticData1=moduleColorsAutomatic1

modules=paste(probes,net1$colors,moduleColorsAutomatic1,sep=",")
write.csv(modules, file = "6-Modules.csv", sep="," ,quote = FALSE,
row.names = FALSE)
##############



############## Normal vs Severe
multiColor=list(Data1=moduleColorsAutomaticData1)
setLabels = c("Data1", "Data2")
multiExpr=list(Data1=list(data=datExpr_n), Data2=list(data=datExpr_s))

nPermutations1=200
```

```
set.seed(1)
system.time({ mp_s = modulePreservation(multiExpr,
multiColor,networkType= "signed",corFnc= "bicor", referenceNetworks = 1,
nPermutations = nPermutations1, randomSeed = 1, quickCor = 0,
maxModuleSize=5000, verbose = 3) })
save(mp_s, file = "7-modulePreservation_s.RData")
##############



############## Mmodule preservation results  ## Excel

# specify the reference and the test networks
ref=1; test = 2

statsObs= cbind(mp_s$quality$observed[[ref]][[test]][,-
1],mp_s$preservation$observed[[ref]][[test]][,-1])
statsZ= cbind(mp_s$quality$Z[[ref]][[test]][,-
1],mp_s$preservation$Z[[ref]][[test]][,-1]);
moduleSize=mp_s$preservation$Z[[ref]][[test]]$moduleSize
log.p=mp_s$preservation$log.p[[ref]][[test]][,-1]


QualityStats=print( cbind(moduleSize ,statsObs[, c("medianRank.pres",
"medianRank.qual")],signif(statsZ[, c("Zsummary.pres",
"Zsummary.qual")],2),signif(log.p[,c("log.psummary.pres")],2)))
##############




############## Mmodule preservation results  ## Plot
Obs.PreservationStats= mp_s$preservation$observed[[ref]][[test]]
Z.PreservationStats=mp_s$preservation$Z[[ref]][[test]]

modColors = rownames(Obs.PreservationStats)
moduleSize = Obs.PreservationStats$moduleSize

selectModules = !(modColors %in% c("grey", "gold"))
point.label = modColors[selectModules]


medianRank=Obs.PreservationStats$medianRank.pres
Zsummary=Z.PreservationStats$Zsummary.pres
par(mfrow=c(1,2),mar = c(4.5,4.5,2.5,1))

plot(moduleSize[selectModules],medianRank[selectModules],col=1,
bg=modColors[selectModules],pch = 21,main="MedianRank Preservation", cex
= 2, ylab ="MedianRank",xlab="Module size", log="x")
labelPoints(moduleSize[selectModules],medianRank[selectModules],point.lab
el,cex=1,offs=0.03)
abline(h=8, col = "red", lty = 2);
```

```
plot(moduleSize[selectModules],Zsummary[selectModules], col = 1,
bg=modColors[selectModules],pch = 21,main="Zsummary preservation",
cex=2,ylab ="Zsummary", xlab = "Module size", log = "x")
labelPoints(moduleSize[selectModules],Zsummary[selectModules],point.label
,cex=1,offs=0.03)
abline(h=5, col = "red", lty = 2)

##############
```

```
############## Normal vs Mild
multiColor=list(Data1=moduleColorsAutomaticData1)
setLabels = c("Data1", "Data2")
multiExpr=list(Data1=list(data=datExpr_n), Data2=list(data=datExpr_m))

nPermutations1=200

set.seed(1)
system.time({ mp_m = modulePreservation(multiExpr, multiColor,
networkType= "signed",corFnc= "bicor", referenceNetworks = 1,
nPermutations = nPermutations1, randomSeed = 1, quickCor =
0,maxModuleSize=5000, verbose = 3 ) })
save(mp_m, file = "9-modulePreservation_m.RData")
##############
```

```
############## Mmodule preservation results  ## Excel
ref=1; test = 2

statsObs= cbind(mp_m$quality$observed[[ref]][[test]][,-
1],mp_m$preservation$observed[[ref]][[test]][,-1])
statsZ= cbind(mp_m$quality$Z[[ref]][[test]][,-
1],mp_m$preservation$Z[[ref]][[test]][,-1]);
moduleSize=mp_m$preservation$Z[[ref]][[test]]$moduleSize
log.p=mp_m$preservation$log.p[[ref]][[test]][,-1]

QualityStats=print( cbind(moduleSize ,statsObs[, c("medianRank.pres",
"medianRank.qual")],signif(statsZ[, c("Zsummary.pres",
"Zsummary.qual")],2),signif(log.p[,c("log.psummary.pres")],2)))
##############
```

```
############## Mmodule preservation results  ## Plot
Obs.PreservationStats= mp_m$preservation$observed[[ref]][[test]]
Z.PreservationStats=mp_m$preservation$Z[[ref]][[test]]
modColors = rownames(Obs.PreservationStats)
moduleSize = Obs.PreservationStats$moduleSize
```

```
selectModules = !(modColors %in% c("grey", "gold"))
point.label = modColors[selectModules]

medianRank=Obs.PreservationStats$medianRank.pres
Zsummary=Z.PreservationStats$Zsummary.pres
par(mfrow=c(1,2),mar = c(4.5,4.5,2.5,1))
plot(moduleSize[selectModules],medianRank[selectModules],col=1,
bg=modColors[selectModules],pch = 21,main="MedianRank Preservation", cex
= 2, ylab ="MedianRank",xlab="Module size", log="x")
labelPoints(moduleSize[selectModules],medianRank[selectModules],point.lab
el,cex=1,offs=0.03)
abline(h=8, col = "red", lty = 2);
plot(moduleSize[selectModules],Zsummary[selectModules], col = 1,
bg=modColors[selectModules],pch = 21,main="Zsummary Preservation",
cex=2,ylab ="Zsummary", xlab = "Module size", log = "x")
labelPoints(moduleSize[selectModules],Zsummary[selectModules],point.label
,cex=1,offs=0.03)
abline(h=5, col = "red", lty = 2)

##############






############## Module membership analysis, kME

moduleLabelsAutomatic1 = net1$colors
moduleColorsAutomatic1 = labels2colors(moduleLabelsAutomatic1)
moduleColorsAutomaticData1=moduleColorsAutomatic1

load(file ="4-datExpr_n.RData")
load(file ="4-datExpr_s.RData")
load(file ="4-datExpr_m.RData")


ME.Data1=moduleEigengenes(datExpr_n,moduleColorsAutomaticData1)$eigengene
s
ME.Data2=moduleEigengenes(datExpr_s,moduleColorsAutomaticData1)$eigengene
s
ME.Data3=moduleEigengenes(datExpr_m,moduleColorsAutomaticData1)$eigengene
s


kME_Normal=signedKME(datExpr_n,ME.Data1,corFnc = "bicor")
kME_Severe=signedKME(datExpr_s,ME.Data2,corFnc = "bicor")
kME_Mild=signedKME(datExpr_m,ME.Data3,corFnc = "bicor")


genename = names(datExpr_n)
```

```
kME_red=as.data.frame(cbind(kME_Normal$kMEred,kME_Severe$kMEred,kME_Mild$
kMEred))
colnames(kME_red)= c("kME_Normal","kME_Severe","kME_Mild")
row.names(kME_red)=names(datExpr_n)
inModule = (moduleColorsAutomaticData1=="red")
kME_red=kME_red[inModule,]


# Plot between different treatments
par(mfrow=c(5,6))
par(mar=c(2,4,3,2))     #c(bottom, left, top, right)

verboseScatterplot(kME_red$kME_Normal, kME_red$kME_Severe,corFnc =
"cor",xlab="Normal", ylab="Severe", abline
=TRUE,abline.color=2,abline.lty =5,  font=1,font.lab=1,font.main=1)
verboseScatterplot(kME_red$kME_Normal, kME_red$kME_Mild,corFnc =
"cor",xlab="Normal", ylab="Mild", abline =TRUE,abline.color=2,abline.lty
=5,  font=1,font.lab=1,font.main=1)
verboseScatterplot(kME_red$kME_Severe, kME_red$kME_Mild,corFnc =
"cor",xlab="Mild", ylab="Severe", abline =TRUE,abline.color=2,abline.lty
=5,  font=1,font.lab=1,font.main=1)

title("Module membership for turquoise module", outer=TRUE,line = -1)



par (mfrow=c(1,1), mar=c(3.5, 3.5, 2, 1), mgp=c(2.4, 0.8, 0), las=1)

ME_pink=as.data.frame(cbind(ME.Data1$MEpink,ME.Data2$MEpink,ME.Data3$MEpi
nk))
colnames(ME_pink)= c("ME_Normal","ME_Severe","ME_Mild")


##############



############## Intramodular connectivity (kIM)

kIM_Normal=intramodularConnectivity.fromExpr(datExpr_n,
moduleColorsAutomaticData1,corFnc = "bicor",networkType = "signed",
scaleByMax=TRUE,power=13)$kWithin
kIM_Severe=intramodularConnectivity.fromExpr(datExpr_s,
moduleColorsAutomaticData1,corFnc = "bicor",networkType = "signed",
scaleByMax=TRUE,power=16)$kWithin
kIM_Mild=intramodularConnectivity.fromExpr(datExpr_m,
moduleColorsAutomaticData1,corFnc = "bicor",networkType = "signed",
scaleByMax=TRUE,power=30)$kWithin



kIM=as.data.frame(cbind(kIM_Normal, kIM_Severe, kIM_Mild))
```

```
colnames(kIM)= c("kIM_Normal","kIM_Severe","kIM_Mild")
row.names(kIM)=names(datExpr_n)



inModule = (moduleColorsAutomaticData1=="yellow")
kIM_yellow=kIM[inModule,]
write.table(kIM_yellow,"kIM_yellow.txt")




par(mfrow=c(1,3))
par(mar=c(8,4,8,4))     #c(bottom, left, top, right)

verboseScatterplot(kIM_green$kIM_Normal,kIM_green$kIM_Severe,corFnc =
"bicor",xlab="Normal", ylab="Severe", abline
=TRUE,abline.color=2,abline.lty =5)
verboseScatterplot(kIM_green$kIM_Normal,kIM_green$kIM_Mild,corFnc =
"bicor",xlab="Normal", ylab="Mild", abline
=TRUE,abline.color=2,abline.lty =5)
verboseScatterplot(kIM_green$kIM_Mild,kIM_green$kIM_Severe,corFnc =
"bicor",xlab="Mild", ylab="Severe", abline
=TRUE,abline.color=2,abline.lty =5)
title("Intramodular connectivity for green module", outer=TRUE,line = -1)




##############
```
