# Supplementary material for: Weighted Gene Co-expression Network Analysis of Endometriosis and Identification of Functional Modules Associated With Its Main Hallmarks
Source: Front Genet. 2018 Oct 12;9:453. doi: 10.3389/fgene.2018.00453 (PMC6194152; doi:10.3389/fgene.2018.00453)
Supplement: FILE S2 — List of the primers used for RT-PCR experiments. [file Data_Sheet_2.pdf]

Supplementary Table 1: List of the primers used for RT-PCR experiments.

	Name	Sequence (5' ⟶ 3')	Amplicon size (bp)
1	STX18	F: cagactccaagagatattcacg<br>R:cacatcacgaggaagaagag	176
2	IGF-1	F: gtgacattgctctcaacatctc<br>R:aattgggttggaagactgct	128
3	IER3	F: ggcaaagtaggagaagaaatgg<br>R: acacagtagacagacggag	133
4	CYP11A1	F:ccaacccagaacgattcct<br>R:ctgccgacttcttcaacag	89
5	CC2D2A	F:gattagacaagaggagaagtgg<br>R:tttcccaactttgccatagg	81
6	AEBP1	F:gactactatgatggtgcgtg<br>R:aaatcgtcatggatgctgg	130
7	HOXB6	F:acggttcaatggtagattcgc<br>R:gtagtatgtgctccttccagtg	114
8	MMP2	F: ggaaagatgtggtgtgcga<br>R:cttggtgtaggtgtaaatggg	177
